# Supplementary material for: Spatiotemporal control of photochromic upconversion through interfacial energy transfer
Source: Nat Commun. 2024 Mar 1;15:1923. doi: 10.1038/s41467-024-46228-5 (PMC10907698; doi:10.1038/s41467-024-46228-5)
Supplement: Supplementary file 1 — Supplementary Information [file 41467_2024_46228_MOESM1_ESM.pdf]

Supplementary Information

Spatiotemporal control of photochromic upconversion through interfacial energy transfer

Long Yan,[1] Jinshu Huang,[1] Zhengce An,[1] Qinyuan Zhang[1] and Bo Zhou[1,*]

[1]State Key Laboratory of Luminescent Materials and Devices, Guangdong Provincial Key Laboratory of Fiber Laser Materials and Applied Techniques, and Guangdong Engineering Technology Research Center of Special Optical Fiber Materials and Devices, South China University of Technology, Guangzhou, 510641, China

E-mail: (BZ) zhoubo@scut.edu.cn

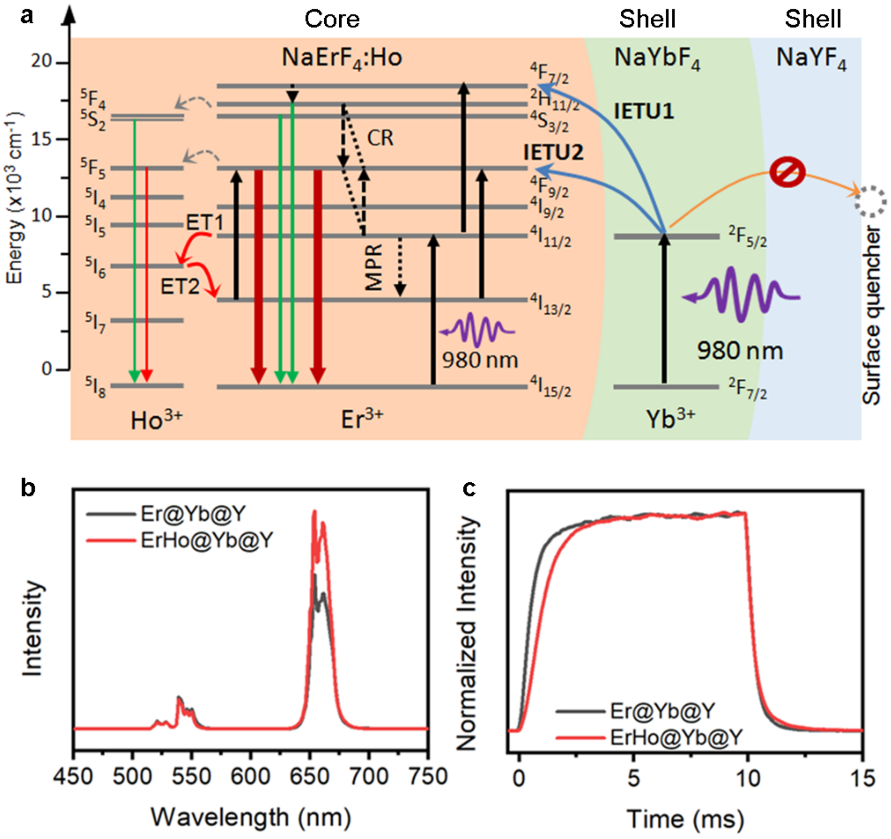

Suppl. Fig. 1 Schematic of enhancing red upconversion of Er^{3+} through Ho^{3+} doping. a Upconversion processes and energy transfer looping (ET1+ET2) from Er^{3+} ($^4I_{11/2}$) to Ho^{3+} ($^5I_6$) and then back to Er^{3+} ($^4I_{13/2}$) for redder emission of Er^{3+} in the proposed design of $NaErF_4$:Ho(0.5 mol%)@$NaYbF_4$@$NaYF_4$ core-shell-shell nanostructure upon 980 nm excitation. **b** Upconversion emission spectra of $NaErF_4$:Ho(0.5 mol%)@$NaYbF_4$@$NaYF_4$ (ErHo@Yb@Y) and $NaErF_4$@$NaYbF_4$@$NaYF_4$ (Er@Yb@Y) core-shell-shell nanoparticles under 980 nm excitation. **c** Time-dependent emission profiles of Er^{3+} at 653 nm from **b** samples.

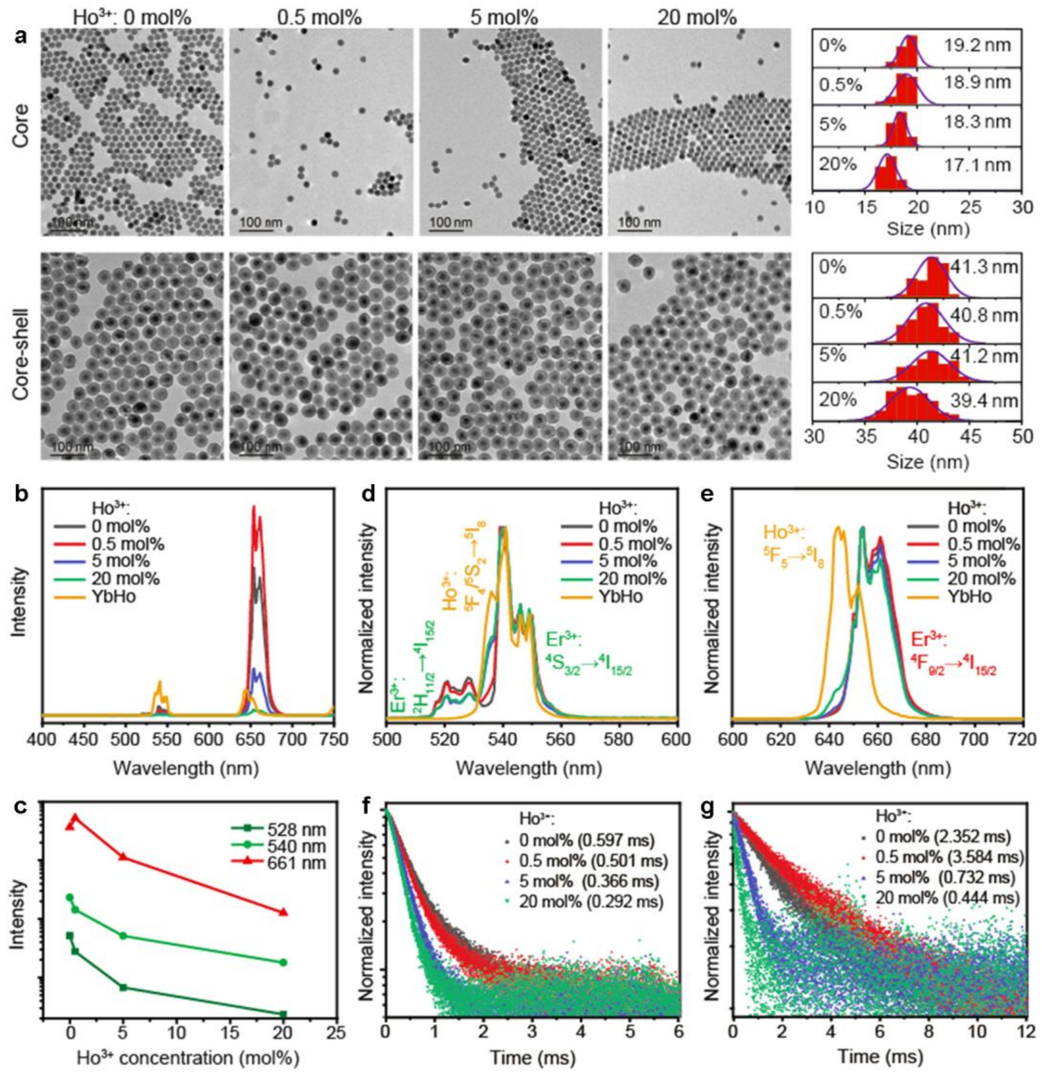

Suppl. Fig. 2 The role of Ho^{3+} in the upconversion of Er^{3+}. a TEM images of $NaErF_4$:Ho(0,0.5,5,20 mol%) core and $NaErF_4$:Ho(0,0.5,5,20 mol%)@$NaYF_4$ core-shell nanoparticles and corresponding size distributions. More than 50 particles in each TEM image were included for statistical analysis. **b** Upconversion emission spectra of **a** samples and the control $NaYF_4$:Yb/Ho(20/2 mol%)@$NaYF_4$ (YbHo) core-shell nanoparticles under 980 nm excitation. **c** Dependence of upconversion emission intensity on Ho^{3+} doping concentration from **b** samples. **d**,**e** A comparison of the normalized upconversion emission spectra of **b** samples under 980 nm excitation, showing that energy transfers between the red and green emission energy levels has no contribution to the redder emission of the sample when Ho^{3+} dopant is as low as 0.5 mol%. **f**,**g** Decay curves of Er^{3+} at its $^4I_{11/2}$ and $^4I_{13/2}$ levels from **a** samples under pulse 980 nm excitation.

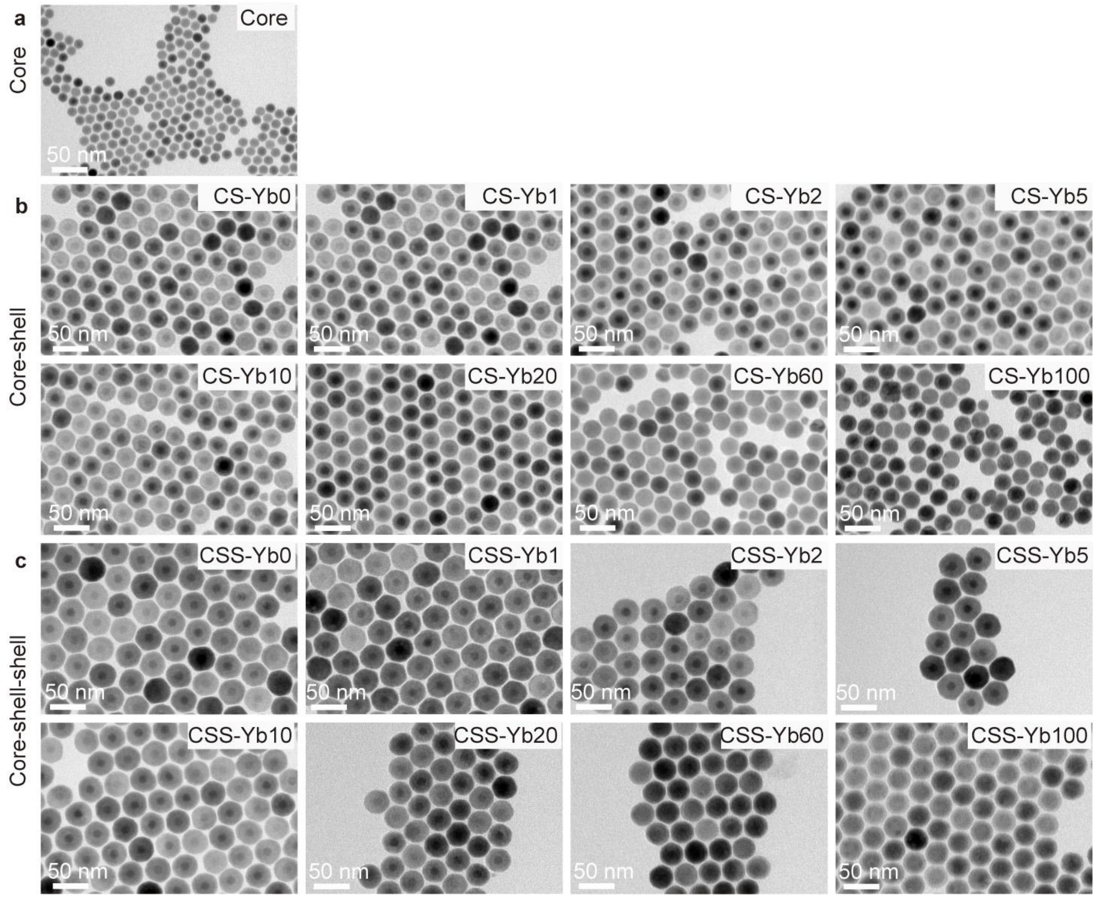

Suppl. Fig. 3 TEM images of $NaErF_4$:Ho(0.5 mol%)@$NaYF_4$:Yb(0-100 mol%)@$NaYF_4$ core-shell-shell nanoparticles at each stage. **a** $NaErF_4$:Ho(0.5 mol%) core nanoparticles. **b** $NaErF_4$:Ho(0.5 mol%)@$NaYF_4$:Yb(0-100 mol%) core-shell nanoparticles. **c** $NaErF_4$:Ho(0.5 mol%)@$NaYF_4$:Yb(0-100 mol%)@$NaYF_4$ core-shell-shell nanoparticles.

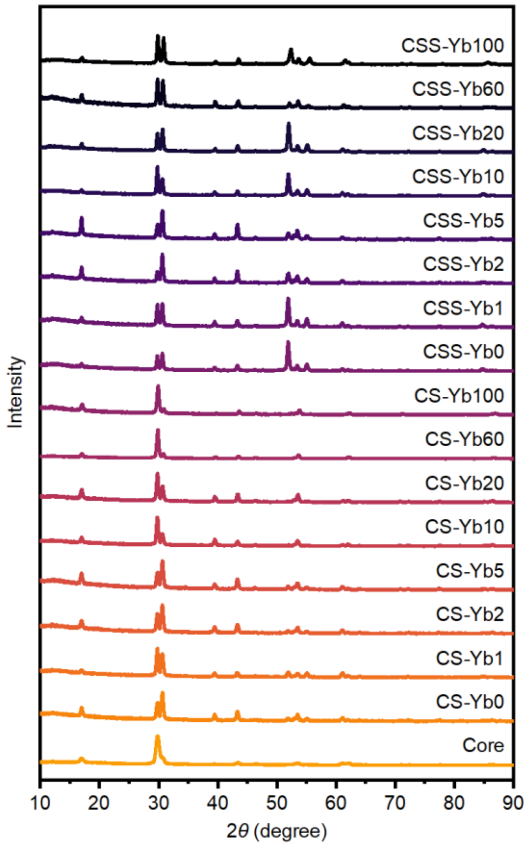

Suppl. Fig. 4 XRD patterns of $NaErF_4$:Ho(0.5 mol%)@$NaYF_4$:Yb(0-100 mol%)@$NaYF_4$ core-shell-shell nanoparticles at each stage. Core represents $NaErF_4$:Ho(0.5 mol%). CS and CSS represent $NaErF_4$:Ho(0.5 mol%)@$NaYF_4$:Yb(0-100 mol%) core-shell and $NaErF_4$:Ho(0.5 mol%)@$NaYF_4$:Yb(0-100 mol%)@$NaYF_4$ core-shell-shell nanoparticles, respectively. The deviation of the diffraction patterns of the nanoparticles were resulted from the random dispersion of them on the surface of silicon slide with different crystalline planes to X-ray.

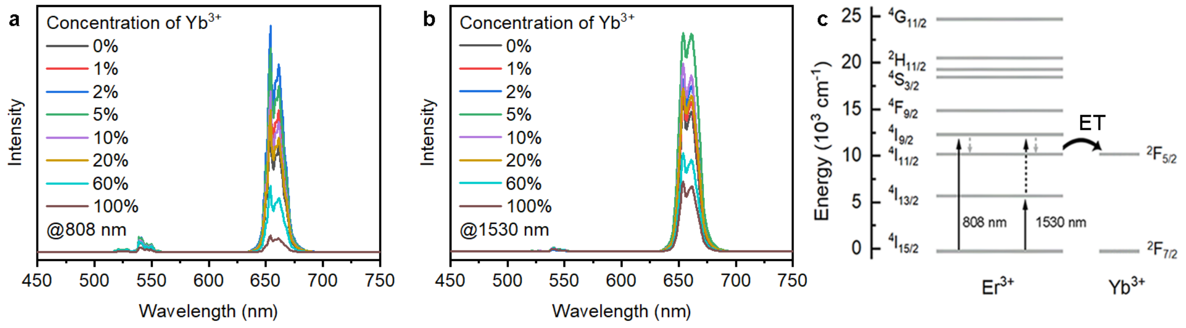

Suppl. Fig. 5 Upconversion emission spectra of $NaErF_4$:Ho(0.5 mol%)@$NaYF_4$:Yb(0-100 mol%)@$NaYF_4$ core-shell-shell nanoparticles under excitation wavelengths of **a** 808 nm and **b** 1530 nm, respectively. The excitation power densities were 11.6 W cm^{-2}. **c** Schematic of energy loss channels through energy transfer from Er^{3+} ($^4I_{11/2}$) to Yb^{3+} ($^2F_{5/2}$) with 808 and 1530 nm excitations.

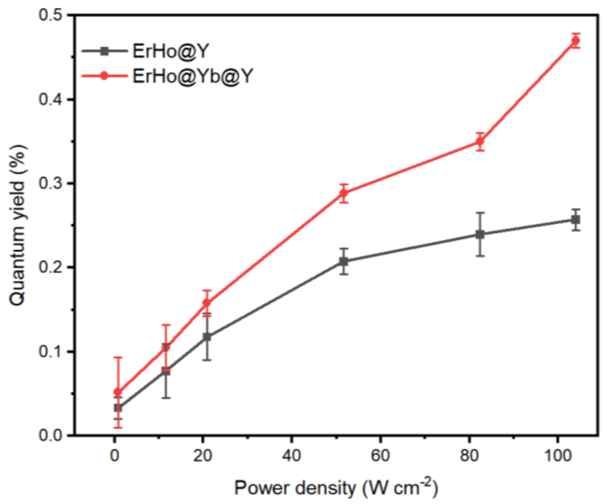

Suppl. Fig. 6 Upconversion quantum yield values obtained from $NaErF_4$:Ho(0.5 mol%)@$NaYbF_4$@$NaYF_4$ (ErHo@Yb@Y) core-shell-shell and $NaErF_4$:Ho(0.5 mol%)@$NaYF_4$ (ErHo@Y) core-shell nanoparticles under 980 nm excitation with variable power densities. Error bars represent the standard deviation of more than 20 trials.

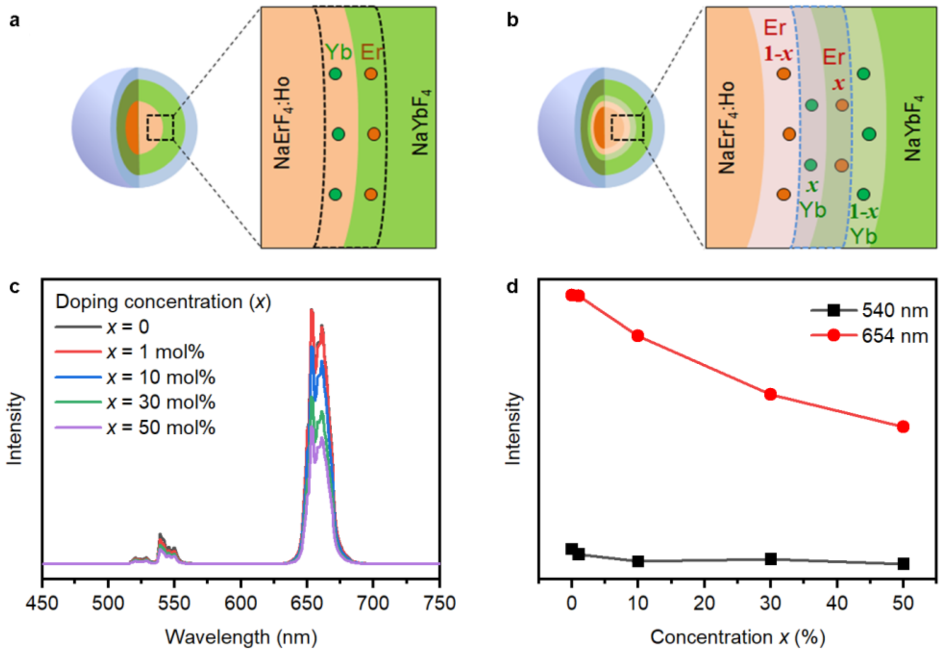

Suppl. Fig. 7 Investigation of possible cation diffusion at interface of the sample. a Schematic of possible ion diffusion at core-shell interfacial region in $NaErF_4$:Ho(0.5 mol%)@$NaYbF_4$@$NaYF_4$ core-shell-shell nanostructure. **b** Proposed $NaErF_4$:Ho(0.5 mol%)@$NaEr_{1-x}Yb_xF_4$@$NaEr_xYb_{1-x}F_4$@$NaYbF_4$@$NaYF_4$ core-multishell nanostructure design to investigate possible ion diffusion by artificially doping identical concentration (x mol%) of Er^{3+} and Yb^{3+} into the newly added interlayers. **c,d** Upconversion emission spectra and upconversion emission intensity of **b** samples with a tuning of doping concentrations (x = 0-50 mol%) under 980 nm excitation.

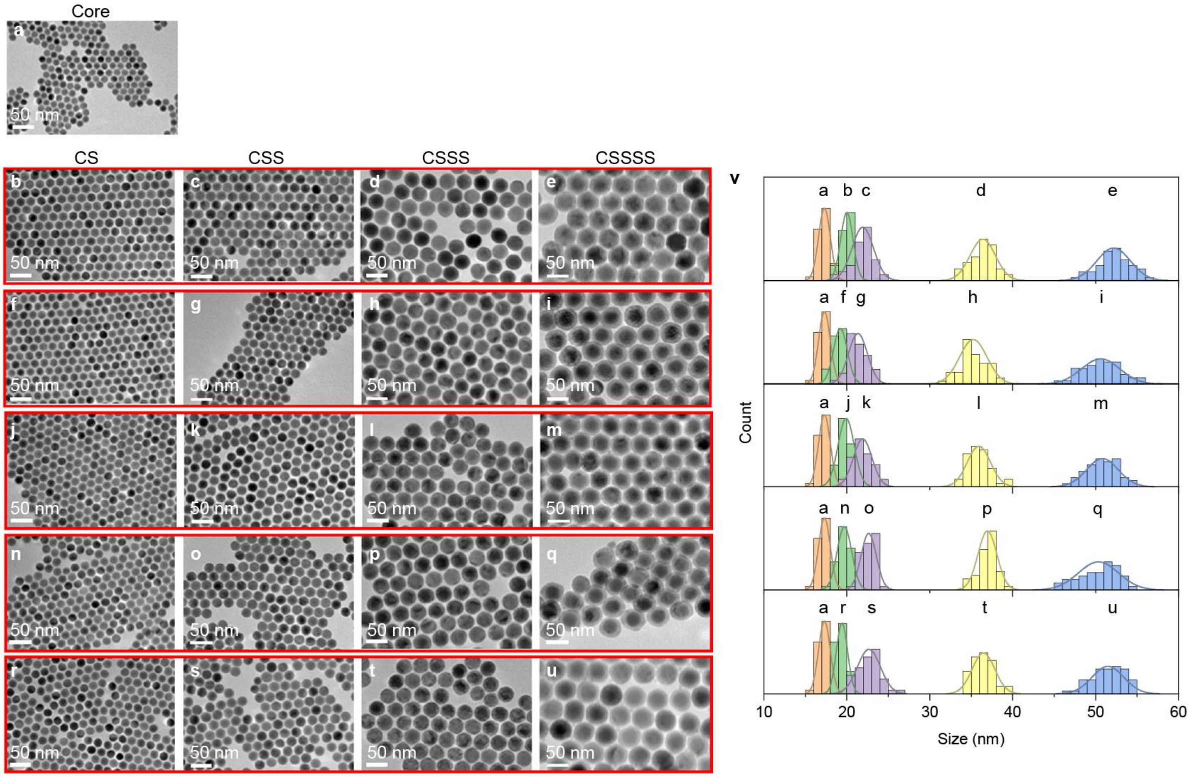

Suppl. Fig. 8 TEM images of **a** $NaErF_4$:Ho(0.5 mol%) core, **b-u** $NaErF_4$:Ho(0.5 mol%)@$NaEr_{1-x}Yb_xF_4$@$NaEr_xYb_{1-x}F_4$@$NaYbF_4$@$NaYF_4$ (x = 0, 1, 10, 30 and 50 mol%) core-multishell nanoparticles at each stage and **v** their particle size distributions. Core presents $NaErF_4$:Ho(0.5 mol%) nanoparticles, CS, CSS, CSSS and CSSSS represent $NaErF_4$:Ho(0.5 mol%)@$NaEr_{1-x}Yb_xF_4$, $NaErF_4$:Ho(0.5 mol%)@$NaEr_{1-x}Yb_xF_4$@$NaEr_xYb_{1-x}F_4$, $NaErF_4$:Ho(0.5 mol%)@$NaEr_{1-x}Yb_xF_4$@$NaEr_xYb_{1-x}F_4$@$NaYbF_4$, and $NaErF_4$:Ho(0.5 mol%)@$NaEr_{1-x}Yb_xF_4$@$NaEr_xYb_{1-x}F_4$@$NaYbF_4$@$NaYF_4$ nanoparticles, respectively. The label above each individual histogram represents the corresponding TEM image number**.** More than 50 particles of each TEM image were included for statistical analysis.

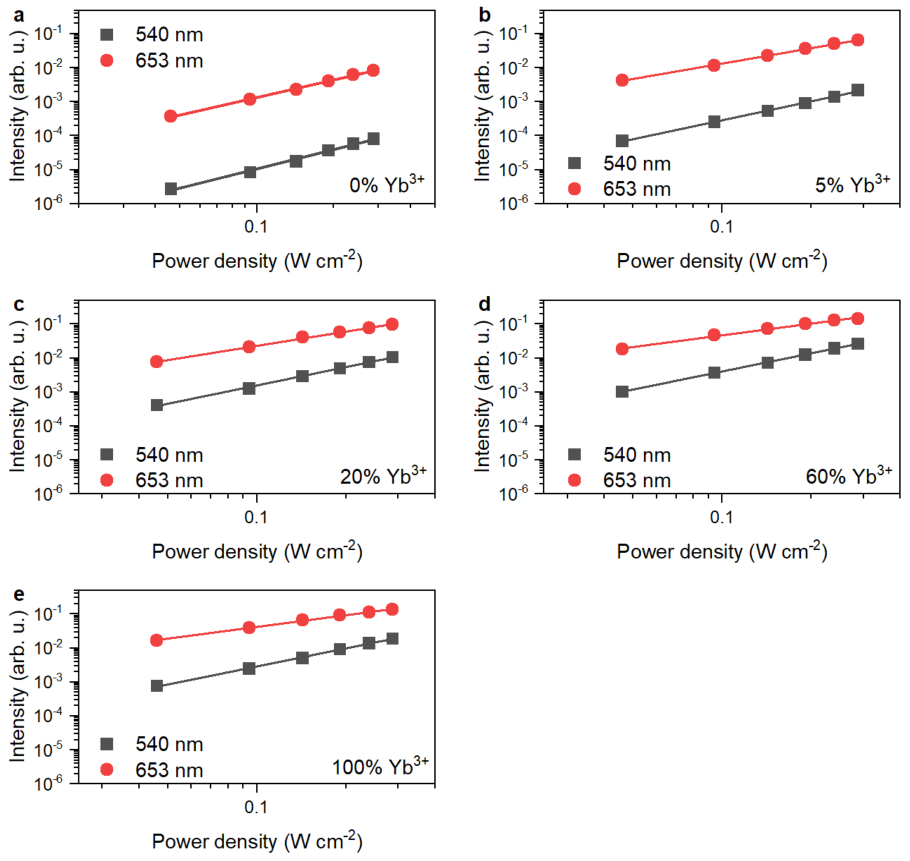

Suppl. Fig. 9 Pump power dependent luminescence intensity of 540 and 653 nm emissions from **a** $NaErF_4$:Ho(0.5 mol%)@$NaYF_4$ core-shell nanoparticles and **b-e** $NaErF_4$:Ho(0.5 mol%)@$NaYF_4$:Yb(5 mol%)@$NaYF_4$, $NaErF_4$:Ho(0.5 mol%)@$NaYF_4$:Yb(20 mol%)@$NaYF_4$, $NaErF_4$:Ho(0.5 mol%)@$NaYF_4$:Yb(60 mol%)@$NaYF_4$, and $NaErF_4$:Ho(0.5 mol%)@$NaYbF_4$@$NaYF_4$ core-shell-shell nanoparticles under 980 nm excitation.

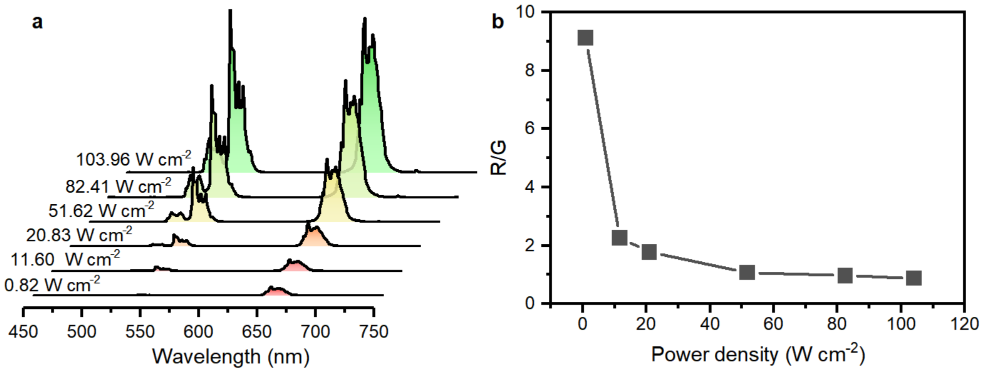

Suppl. Fig. 10 Pump power dependent photochromic upconversion. a Upconversion emission spectra and **b** corresponding red-to-green emission ratio (653 nm / 540 nm, R/G) with variable 980 nm excitation power density for $NaErF_4$:Ho(0.5 mol%)@$NaYF_4$:Yb(20 mol%)@$NaYF_4$ core-shell-shell nanoparticles.

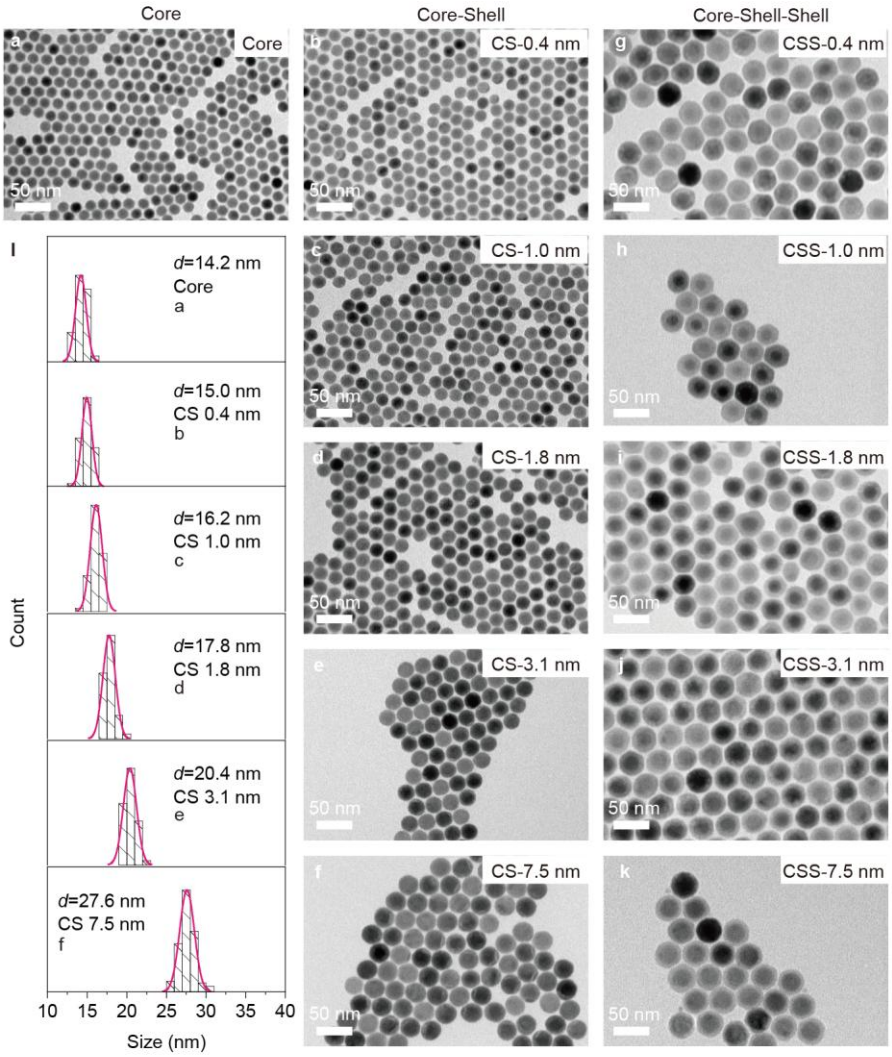

Suppl. Fig. 11 TEM images of **a** $NaErF_4$:Ho(0.5 mol%) core, **b-f** $NaErF_4$:Ho(0.5 mol%)@$NaYbF_4$ core-shell and **g-k** $NaErF_4$:Ho(0.5 mol%)@$NaYbF_4$@$NaYF_4$ core-shell-shell nanoparticles with increasing $NaYbF_4$ layer thickness (0.4-7.5 nm) and **l** their particle size distributions. Core represents $NaErF_4$:Ho(0.5 mol%) nanoparticles, CS and CSS represent $NaErF_4$:Ho(0.5 mol%)@$NaYbF_4$ core-shell nanoparticles, respectively. More than 50 particles of each TEM image were included for statistical analysis.

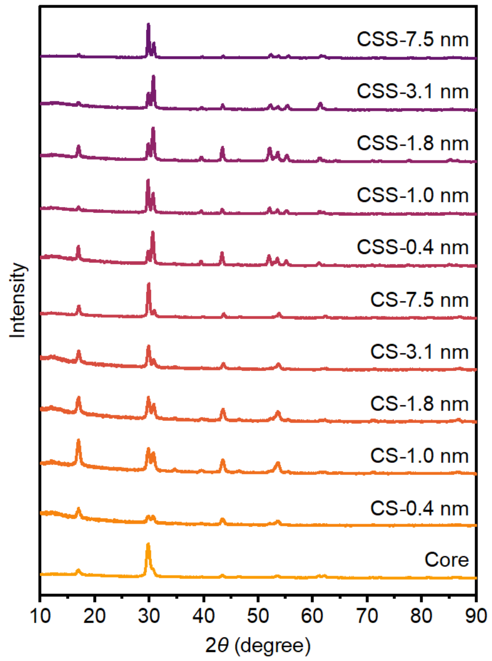

Suppl. Fig. 12 XRD patterns of $NaErF_4$:Ho(0.5 mol%)@$NaYbF_4$@$NaYF_4$ core-shell-shell nanoparticles with increasing $NaYbF_4$ layer thickness (0-7.5 nm). Core represents $NaErF_4$:Ho(0.5 mol%) nanoparticles. CS and CSS represent $NaErF_4$:Ho(0.5 mol%)@$NaYbF_4$ core-shell and $NaErF_4$:Ho(0.5 mol%)@$NaYbF_4$@$NaYF_4$ core-shell-shell nanoparticles.

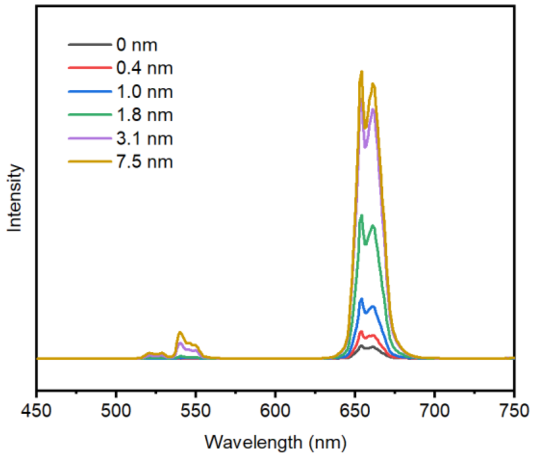

Suppl. Fig. 13 Upconversion emission spectra of $NaErF_4$:Ho(0.5 mol%)@$NaYbF_4$@$NaYF_4$ core-shell-shell nanoparticles with finely tuning the thickness of $NaYbF_4$ layer under 980 nm excitation.

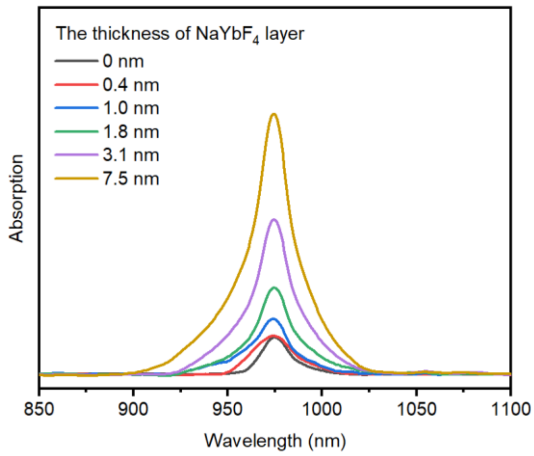

Suppl. Fig. 14 Absorption spectra of $NaErF_4$:Ho(0.5 mol%)@$NaYbF_4$@$NaYF_4$ core-shell-shell nanoparticles with finely tuning the thickness of $NaYbF_4$ layer.

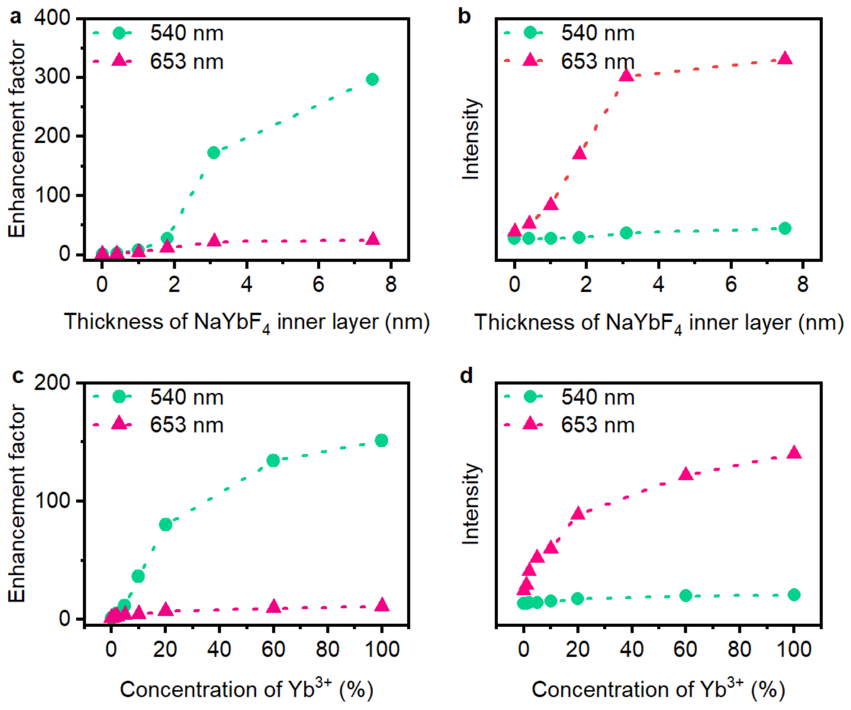

Suppl. Fig. 15 Upconversion versus $NaYbF_4$ layer thickness and Yb^{3+} concentration. a Upconversion enhancement factor and **b** recorded upconversion emission intensity from the $NaErF_4$:Ho(0.5 mol%)@$NaYbF_4$@$NaYF_4$ core-shell-shell nanoparticles with thicknesses of $NaYbF_4$ interlayer from 0 to 7.5 nm. **c** Upconversion enhancement factor and **d** recorded upconversion emission intensity from the $NaErF_4$:Ho(0.5 mol%)@$NaYF_4$:Yb(0-100 mol%)@$NaYF_4$ core-shell-shell nanoparticles with Yb^{3+} concentrations in interlayer from 0 to 100 mol%.

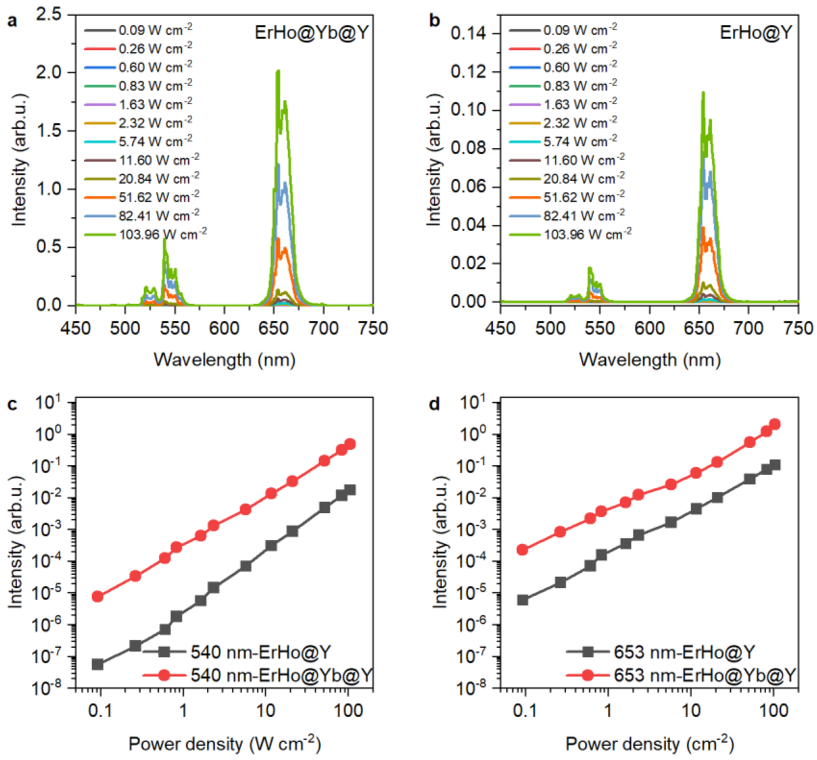

Suppl. Fig. 16 Pump power dependent upconversion. a,**b** Upconversion emission spectra of $NaErF_4$:Ho(0.5 mol%)@$NaYbF_4$@$NaYF_4$ core-shell-shell (ErHo@Yb@Y) and $NaErF_4$:Ho(0.5 mol%)@$NaYF_4$ core-shell (ErHo@Y) nanoparticles under 980 nm excitation at different pump power densities. **c**,**d** Dependence of the upconversion intensity of green and red emissions from **a** and **b** samples.

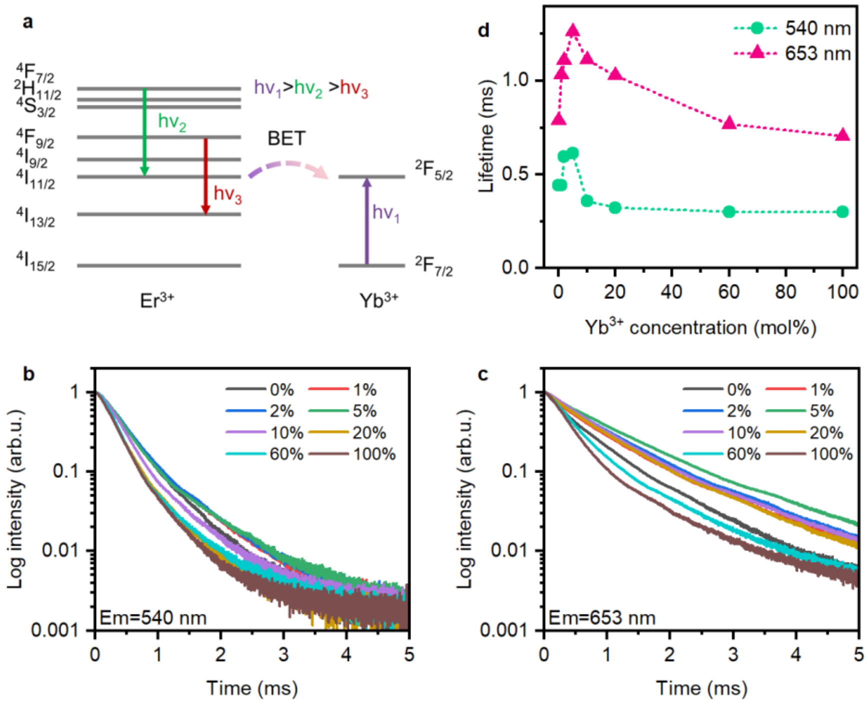

Suppl. Fig. 17 Investigation of BET. a Schematic of possible BET processes from Er^{3+} ($^4F_{7/2}$, $^4F_{9/2}$ and $^4I_{11/2}$) to Yb^{3+}. There is a small mismatch for BET from $^4F_{7/2}$ and $^4F_{9/2}$ states of Er^{3+}, while it is resonant for that from $^4I_{11/2}$ state of Er^{3+}. **b**,**c** Decay curves of Er^{3+} at its $^4S_{3/2}$ (540 nm) and $^4F_{9/2}$ (653 nm) states from $NaErF_4$:Ho(0.5 mol%)@$NaYF_4$:Yb(0-100 mol%)@$NaYF_4$ core-shell-shell nanoparticles upon pulse 980 nm excitation. **d** Lifetime values of Er^{3+} on Yb^{3+} concentrations obtained from **b** and **c** samples.

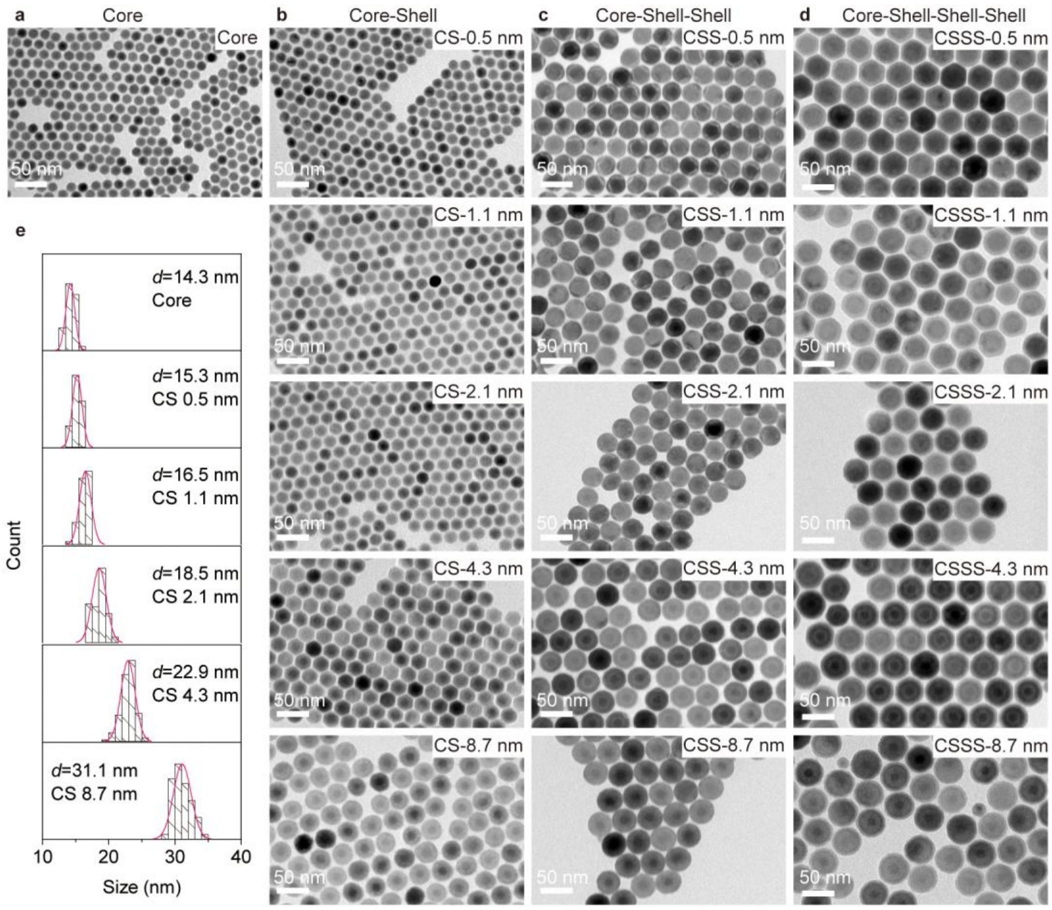

Suppl. Fig. 18 TEM images of **a-d** $NaErF_4$:Ho(0.5 mol%)@$NaYF_4$@$NaYbF_4$@$NaYF_4$ core-multishell nanoparticles with increasing $NaYbF_4$ layer thickness at each stage and **e** thier particle size distributions. C, CS, CSS and CSSS represent the $NaErF_4$:Ho(0.5 mol%) core, $NaErF_4$:Ho(0.5 mol%)@$NaYF_4$ core-shell, $NaErF_4$:Ho(0.5 mol%)@$NaYF_4$@$NaYbF_4$ core-shell-shell and $NaErF_4$:Ho(0.5 mol%)@$NaYF_4$@$NaYbF_4$@$NaYF_4$ core-multishell nanoparticles with different $NaYF_4$ inter layer thicknesses (0.5-8.7 nm), respectively. More than 50 particles of each TEM image were included for statistical analysis.

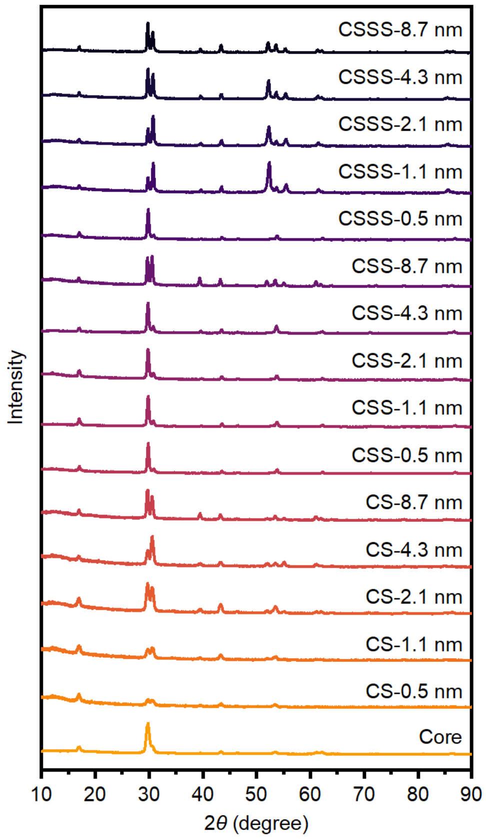

Suppl. Fig. 19 XRD patterns of $NaErF_4$:Ho(0.5 mol%)@$NaYF_4$@$NaYbF_4$@$NaYF_4$ nanoparticles with increasing $NaYbF_4$ layer thickness (0-8.7 nm). Core represents $NaErF_4$:Ho(0.5 mol%) nanoparticles. CS, CSS and CSSS represent $NaErF_4$:Ho(0.5 mol%)@$NaYF_4$ core-shell, $NaErF_4$:Ho(0.5 mol%)@$NaYF_4$@$NaYbF_4$ core-shell-shell and $NaErF_4$:Ho(0.5 mol%)@$NaYF_4$@$NaYbF_4$@ $NaYF_4$ core-multishell nanoparticles.

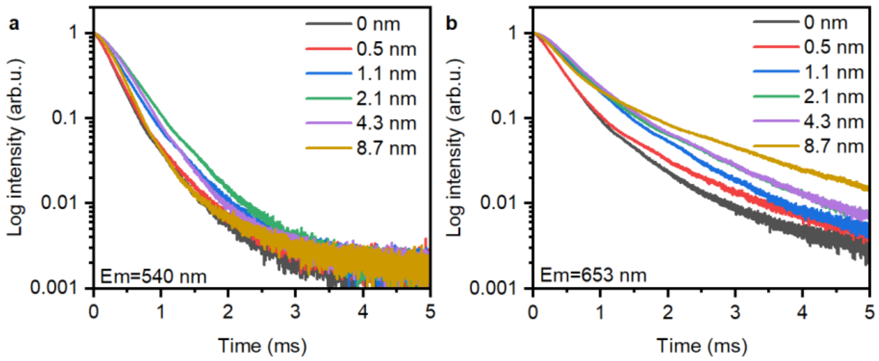

Suppl. Fig. 20 Decay curves of Er^{3+} at its **a** $^4S_{3/2}$ (540 nm) and **b** $^4F_{9/2}$ (653 nm) states from $NaErF_4$:Ho(0.5 mol%)@$NaYF_4$@$NaYbF_4$@$NaYF_4$ core-multishell nanoparticles with increasing $NaYF_4$ interlayer thickness (0-8.7 nm) upon pulse 980 nm excitation.

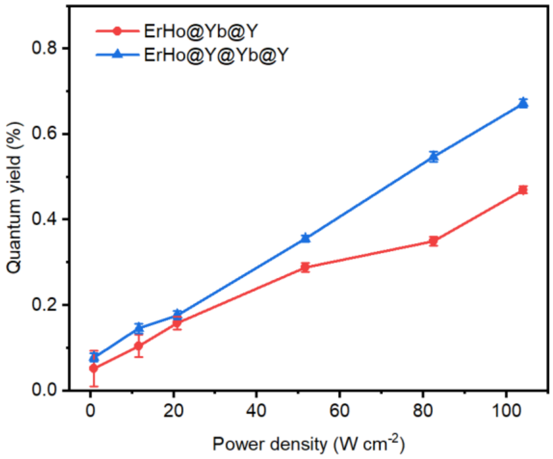

Suppl. Fig. 21 Upconversion quantum yield values obtained from the $NaErF_4$:Ho(0.5 mol%)@$NaYF_4$@$NaYbF_4$@$NaYF_4$ (ErHo@Y@Yb@Y) core-multishell and $NaErF_4$:Ho(0.5 mol%)@$NaYbF_4$@$NaYF_4$ (ErHo@Yb@Y) core-shell-shell nanoparticles under 980 nm excitation with variable power densities. Error bars represent the standard deviation of more than 20 trials.

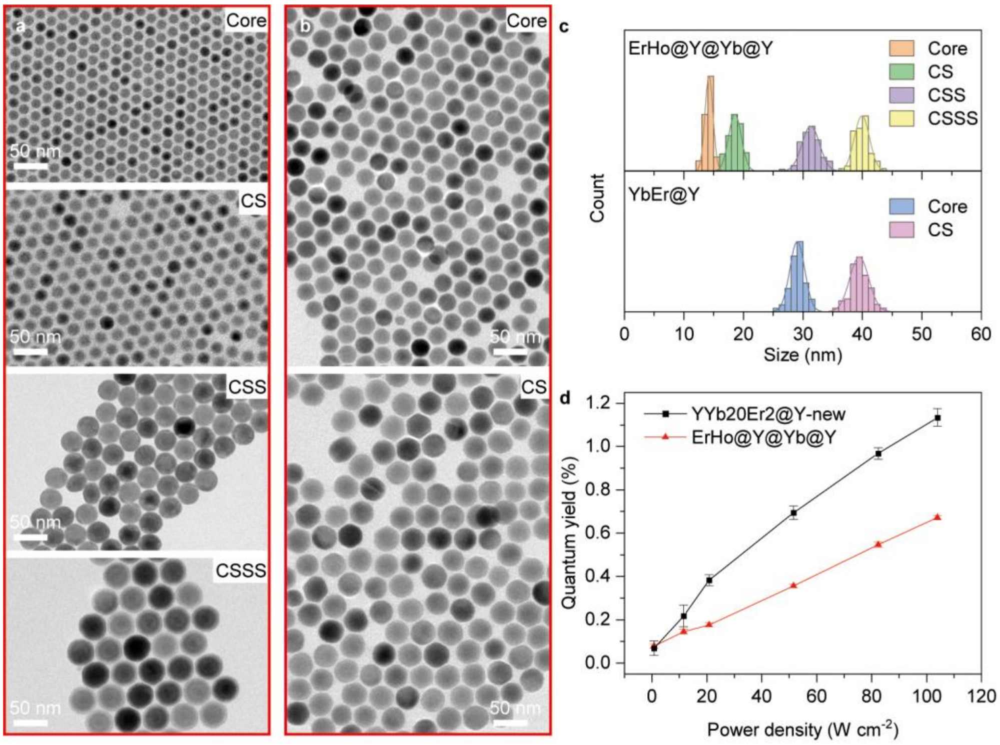

Suppl. Fig. 22 Comparison of quantum yield values. **a**,**b** TEM images of the **a** $NaErF_4$:Ho(0.5 mol%)@$NaYF_4$@$NaYbF_4$@$NaYF_4$ core-multishell (ErHo@Y@Yb@Y) and **b** $NaYF_4$:Yb/Er(20/2 mol%)@$NaYF_4$ core-shell (Yb20Er2@Y) nanoparticles at each stage. **c** The corresponding size distributions of **a** and **b** samples. More than 50 particles of each TEM image were included for statistical analysis. **d** Upconversion quantum yield values obtained from **a** and **b** samples under 980 nm excitation with variable pump power densities. Error bars represent the standard deviation of more than 20 trials.

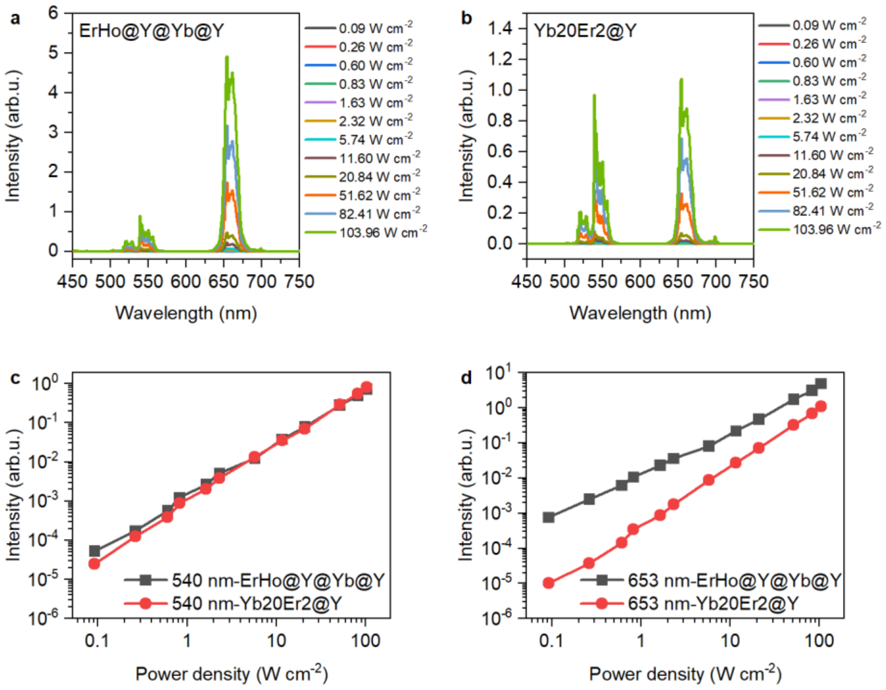

Suppl. Fig. 23 Pump power dependent upconversion. a,**b** Upconversion emission spectra of $NaErF_4$:Ho(0.5 mol%)@$NaYF_4$@$NaYbF_4$@$NaYF_4$ core-multishell (ErHo@Y@Yb@Y) and $NaYF_4$:Yb/Er(20/2 mol%)@$NaYF_4$ core-shell (Yb20Er2@Y) nanoparticles under 980 nm excitation at different pump power densities. **c**,**d** Dependence of the upconversion intensity of green **c** and red **d** emissions from **a** and **b** samples.

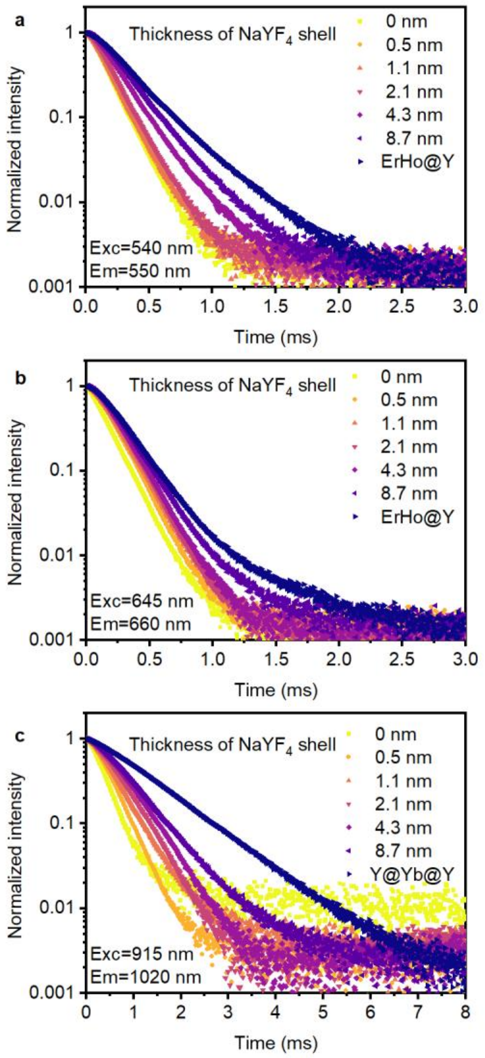

Suppl. Fig. 24 Decay curves of Er^{3+} and Yb^{3+} versus $NaYF_4$ shell thickness. a,b Decay curves of Er^{3+} at its $^4S_{3/2}$ (550 nm) and $^4F_{9/2}$ (660 nm) from $NaErF_4$:Ho(0.5 mol%)@$NaYF_4$@$NaYbF_4$@$NaYF_4$ core-multishell nanoparticles with increasing $NaYF_4$ interlayer thickness (0-8.7 nm) and that from the control sample of $NaYF_4$@$NaYbF_4$@$NaYF_4$ core-shell-shell nanoparticles. **c** Decay curves of Yb^{3+} at its $^2F_{5/2}$ (1020 nm) in $NaErF_4$:Ho(0.5 mol%)@$NaYF_4$@$NaYbF_4$@$NaYF_4$ core-multishell nanoparticles with increasing $NaYF_4$ interlayer thickness (0-8.7 nm) and that from the control sample of $NaYF_4$@$NaYbF_4$@$NaYF_4$ core-shell-shell nanoparticles.

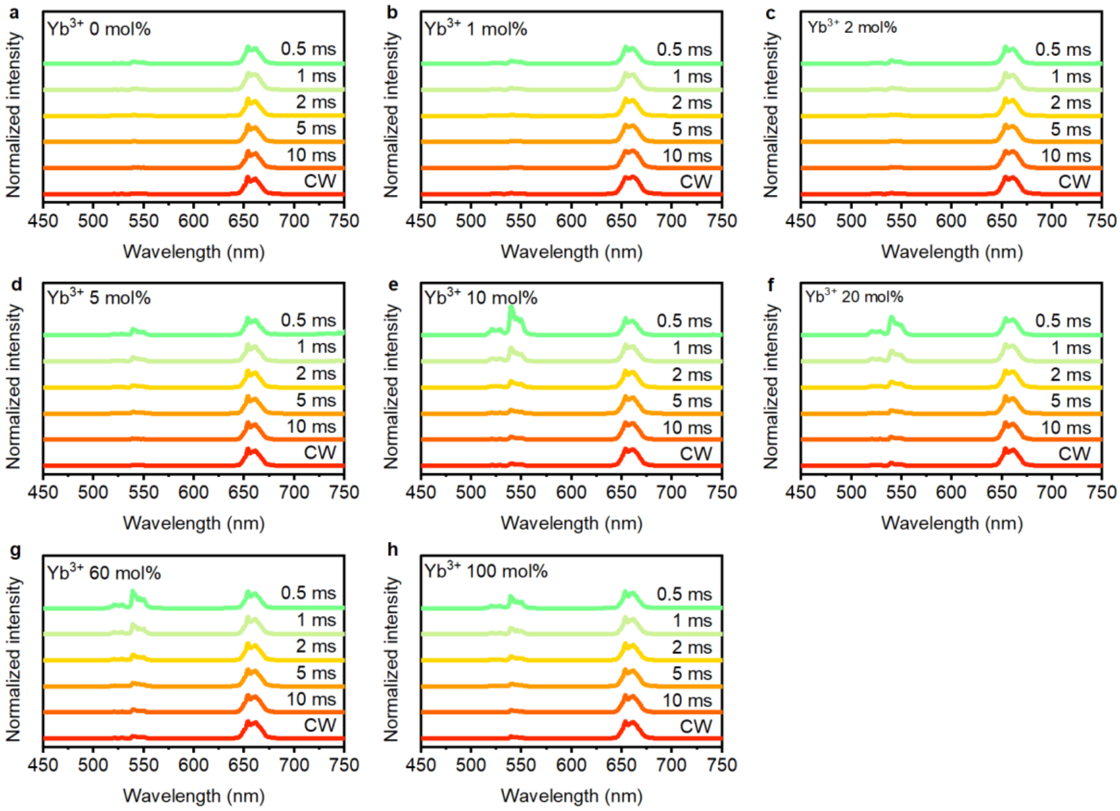

Suppl. Fig. 25 Normalized non-steady state upconversion emission spectra of **a** $NaErF_4$:Ho(0.5 mol%)@$NaYF_4$ core-shell and **b-h** $NaErF_4$:Ho(0.5 mol%)@$NaYF_4$:Yb(1,2,5,10,20,60,100 mol%)@$NaYF_4$ core-shell-shell nanoparticles under pulse 980 nm excitation with frequency of 50 Hz. All emission spectra were normalized to the red emission intensity for an easy comparison.

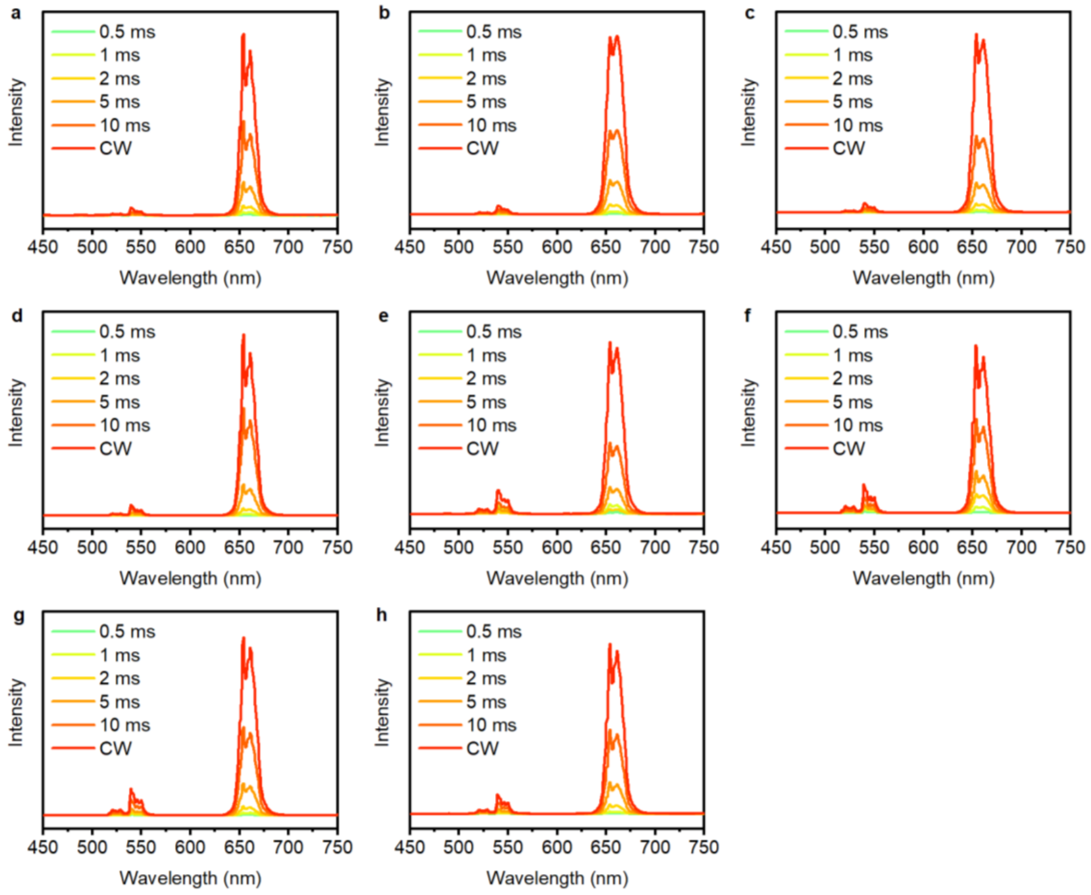

Suppl. Fig. 26 Non-steady state upconversion emission spectra of **a** $NaErF_4$:Ho(0.5 mol%)@$NaYF_4$ core-shell and **b-h** $NaErF_4$:Ho(0.5 mol%)@$NaYF_4$:Yb(1,2,5,10,20,60,100 mol%)@$NaYF_4$ core-shell-shell nanoparticles under pulse 980 nm excitation with frequency of 50 Hz.

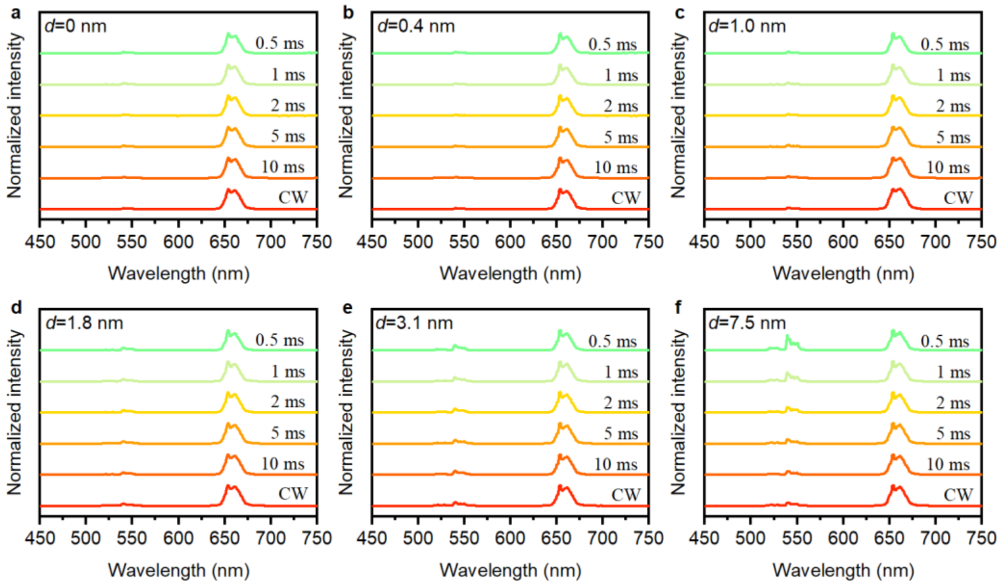

Suppl. Fig. 27 Normalized non-steady state upconversion emission spectra of **a-f** the $NaErF_4$:Ho(0.5 mol%)@$NaYbF_4$@$NaYF_4$ core-shell-shell nanoparticles under pulse 980 nm excitation with frequency of 50 Hz. The thickness of $NaYbF_4$ layer was precisely controlled from 0 to 7.5 nm. All emission spectra were normalized to the red emission intensity for an easy comparison.

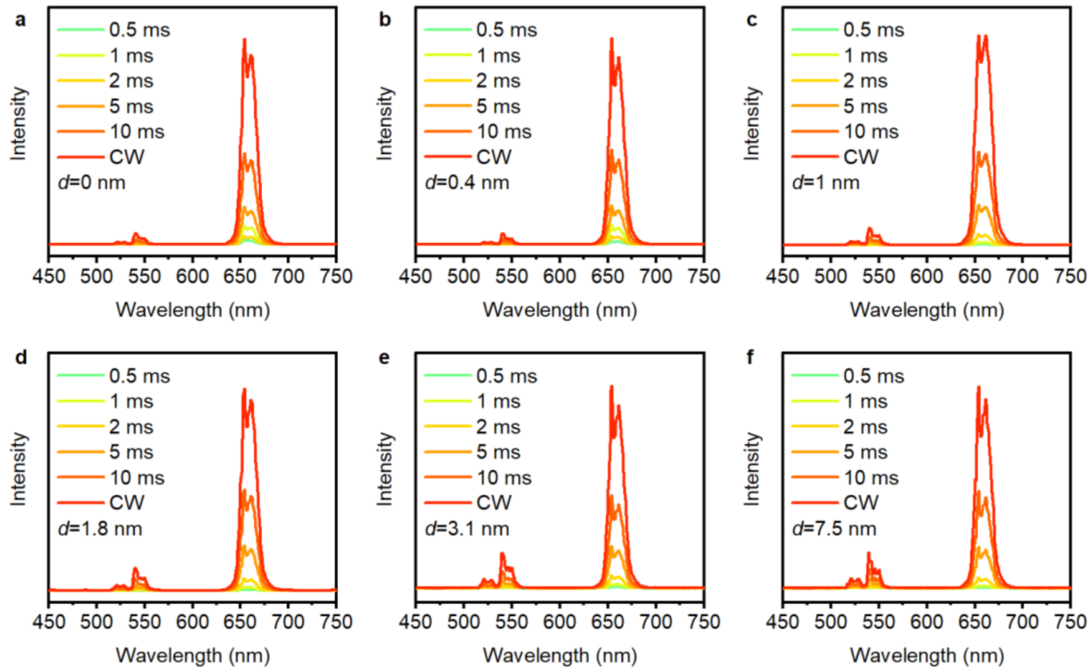

Suppl. Fig. 28 Non-steady state upconversion emission spectra of **a-f** the $NaErF_4$:Ho(0.5 mol%)@$NaYbF_4$@$NaYF_4$ core-shell-shell nanoparticles under pulse 980 nm excitation with frequency of 50 Hz. The thickness of $NaYbF_4$ layer was precisely controlled from 0 to 7.5 nm.

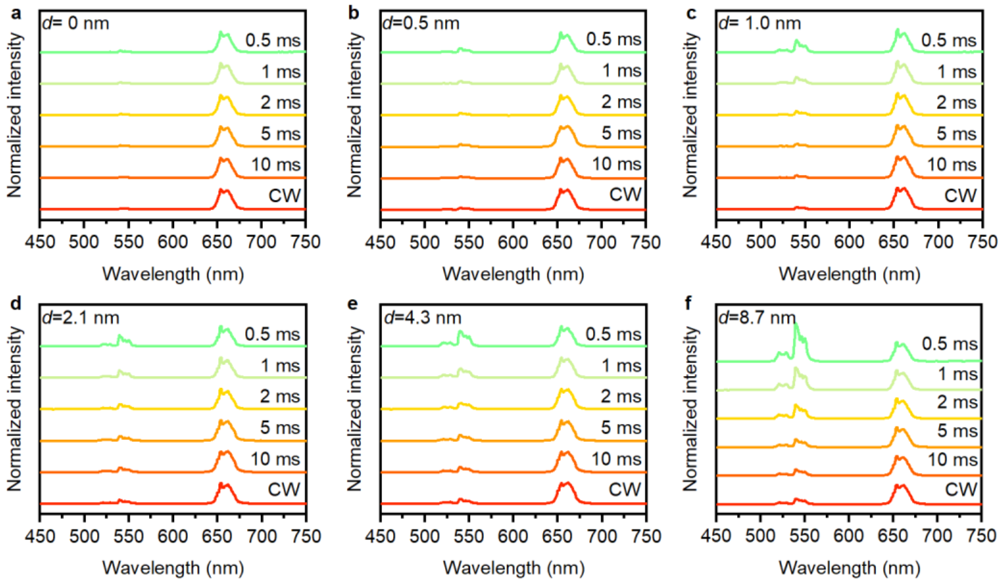

Suppl. Fig. 29 Normalized non-steady state upconversion emission spectra of **a-f** the $NaErF_4$:Ho(0.5 mol%)@$NaYF_4$@$NaYbF_4$@$NaYF_4$ core-shell-shell nanoparticles under pulse 980 nm excitation with 50 Hz. The thickness of $NaYF_4$ interlayer was precisely controlled from 0 to 8.7 nm. All emission spectra were normalized to the red emission intensity for an easy comparison.

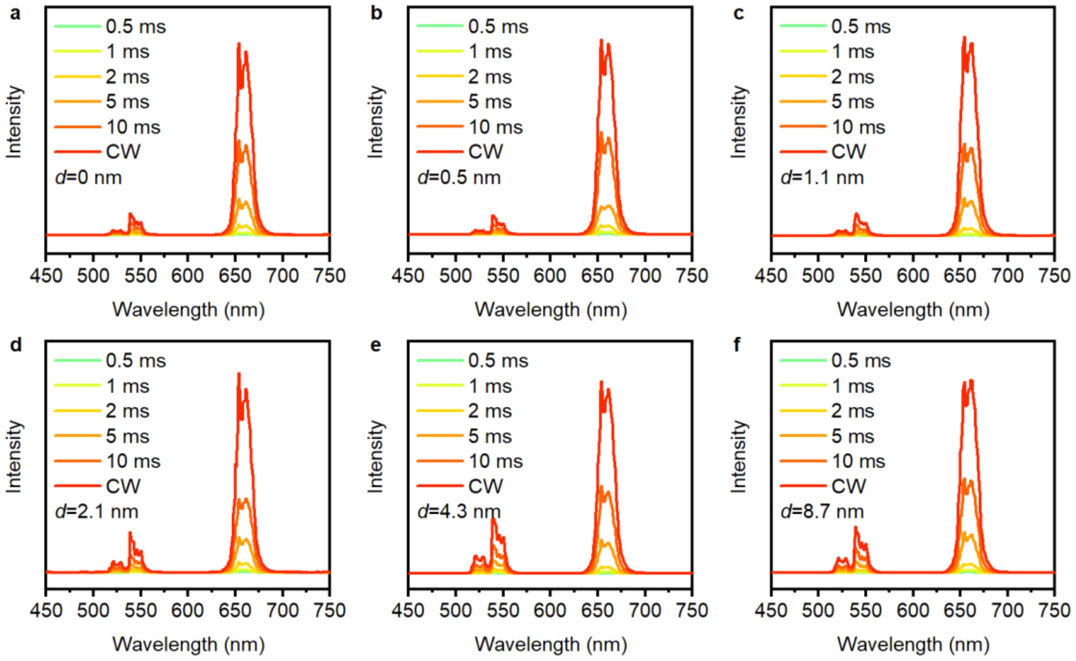

Suppl. Fig. 30 Non-steady state upconversion emission spectra of **a-f** the $NaErF_4$:Ho(0.5 mol%)@$NaYF_4$@$NaYbF_4$@$NaYF_4$ core-shell-shell nanoparticles under pulse 980 nm excitation with frequency of 50 Hz. The thickness of $NaYF_4$ interlayer was precisely controlled from 0 to 8.7 nm.

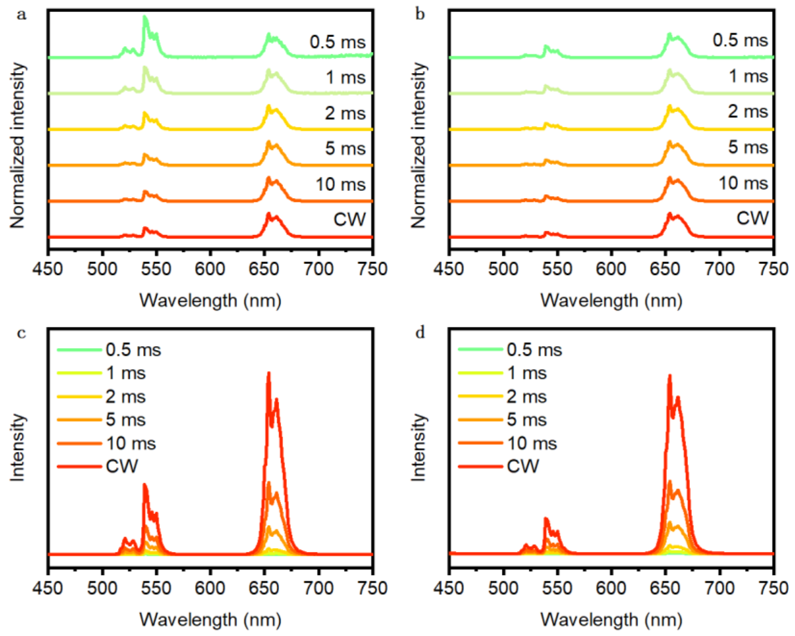

Suppl. Fig. 31 Normalized non-steady state upconversion emission spectra of **a** $NaErF_4$@$NaYF_4$:Yb(20 mol%)@$NaYF_4$ and **b** $NaErF_4$@$NaYbF_4$@$NaYF_4$ core-shell-shell nanoparticles under pulse 980 nm excitation with frequency of 50 Hz. **c**,**d** Corresponding recorded emission spectra of **a** and **b**.

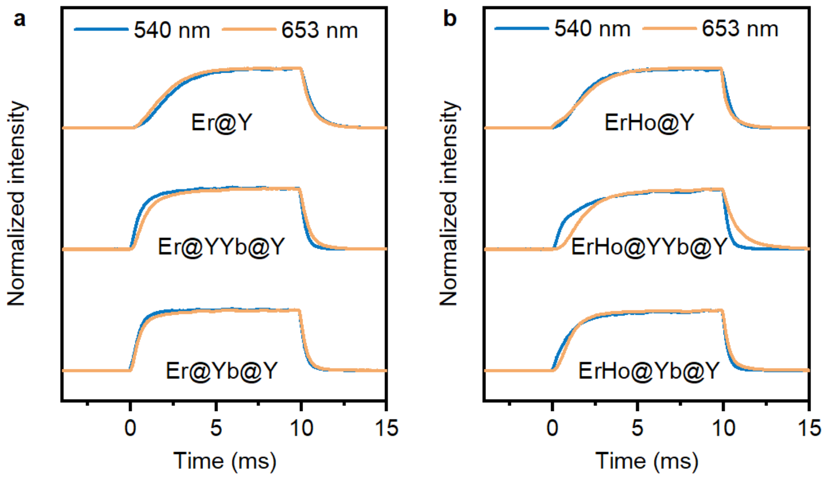

Suppl. Fig. 32 Time-dependent emission profiles of Er^{3+} at 540 and 653 nm from **a** $NaErF_4$@$NaYF_4$ (Er@Y), $NaErF_4$@$NaYF_4$:Yb(20 mol%)@$NaYF_4$ (Er@YYb@Y), and $NaErF_4$@$NaYbF_4$@$NaYF_4$ (Er@Yb@Y) nanoparticles, and **b** $NaErF_4$:Ho(0.5 mol%)@$NaYF_4$ (ErHo@Y), $NaErF_4$:Ho(0.5 mol%)@$NaYF_4$:Yb(20 mol%)@$NaYF_4$ (ErHo@YYb@Y), $NaErF_4$:Ho(0.5 mol%)@$NaYbF_4$@$NaYF_4$ (ErHo@Yb@Y) nanoparticles under 980 nm excitation with pulse duration of 10 ms.

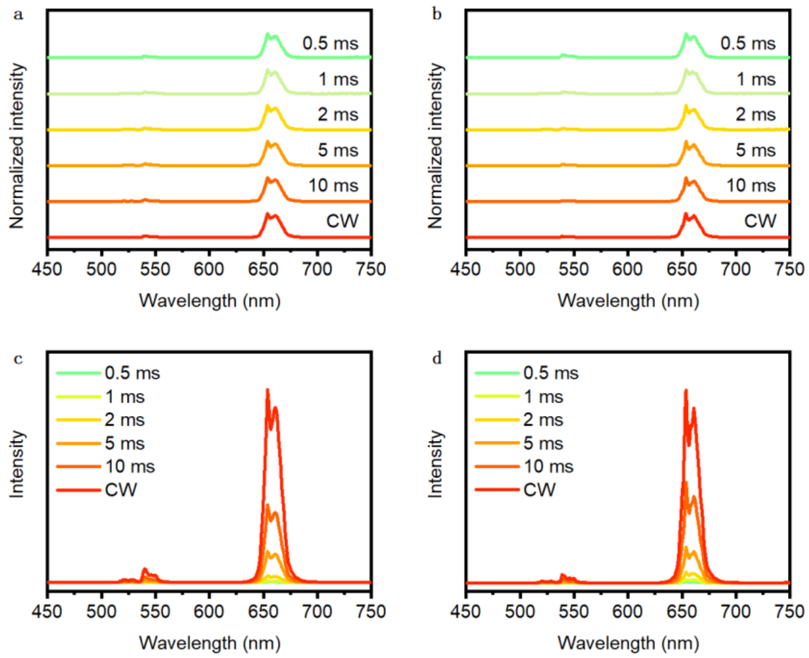

Suppl. Fig. 33 Normalized non-steady state upconversion emission spectra of **a** $NaErF_4@NaYF_4$ and **b** $NaErF_4$:Ho(0.5 mol%)@$NaYF_4$ core-shell nanoparticles under pulse 980 nm excitation with frequency of 50 Hz. **c**,**d** Corresponding recorded emission spectra of **a** and **b**.

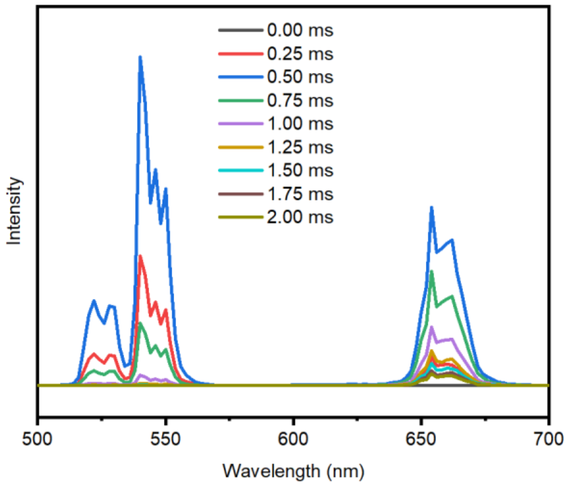

Suppl. Fig. 34 Time-resolved upconversion emission spectra of $NaErF_4$(0.5 mol%)@$NaYF_4$:Yb(20 mol%)@$NaYF_4$ core-shell-shell nanoparticles under pulse 980 nm excitation (Frequency=50 Hz, Width=0.5 ms).

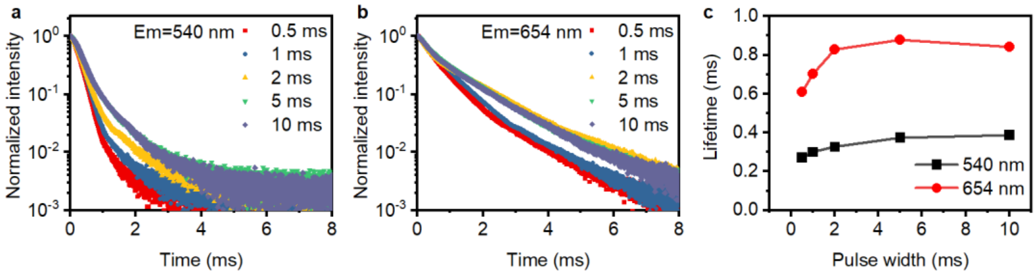

Suppl. Fig. 35 Decay curves of Er^{3+} at its **a** $^4S_{3/2}$ (540 nm) and **b** $^4F_{9/2}$ (653 nm) from $NaErF_4$:Ho(0.5 mol%)@$NaYF_4$:Yb(10 mol%)@$NaYF_4$ core-shell-shell nanoparticles under 980 nm excitation with different pulse widths (0.5-10 ms). **c** Lifetime values obtained from **a** and **b**.

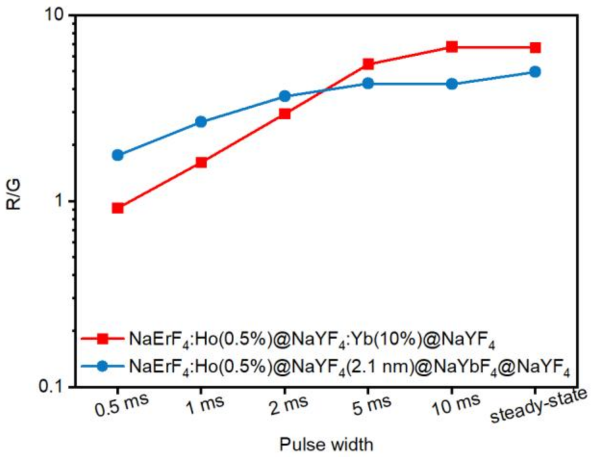

Suppl. Fig. 36 Dependence of red-to-green emission ratio (653 nm / 540 nm, R/G) on pulse widths and CW of 980 nm excitation laser for $NaErF_4$:Ho(0.5%)@$NaYF_4$(2.1 nm)@$NaYbF_4$@$NaYF_4$ and $NaErF_4$:Ho(0.5%)@$NaYF_4$:Yb(20%)@$NaYF_4$ core-multishell nanoparticles.

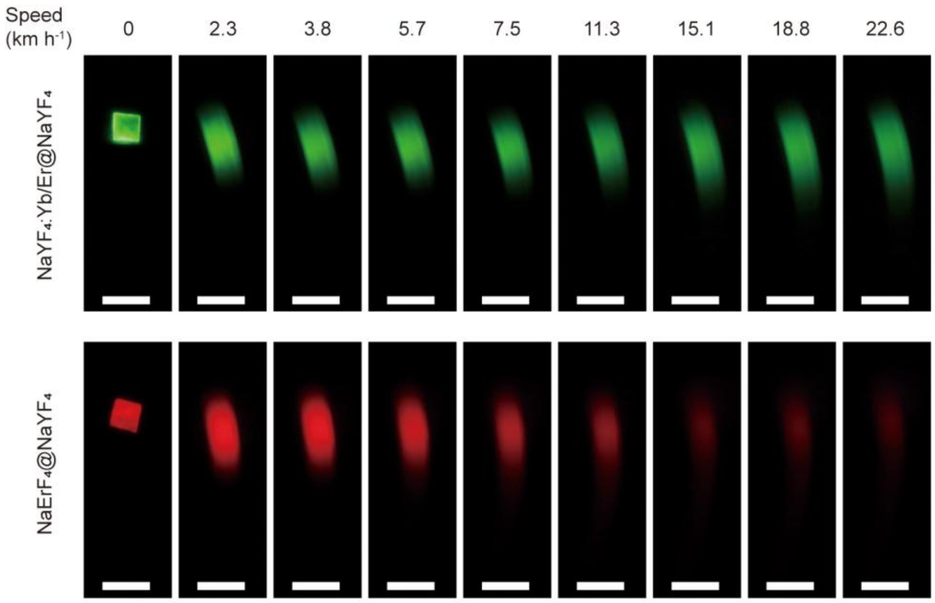

Suppl. Fig. 37 Dynamic light color evolution in $NaYF_4$:Yb/Er(20/2 mol%) and $NaErF_4$@$NaYF_4$ nanoparticles, showing no colour change with increasing rotation speed of the disk under steady-state 980 nm laser excitation. Pattern length is 3 mm. Scale bars, 5 mm.

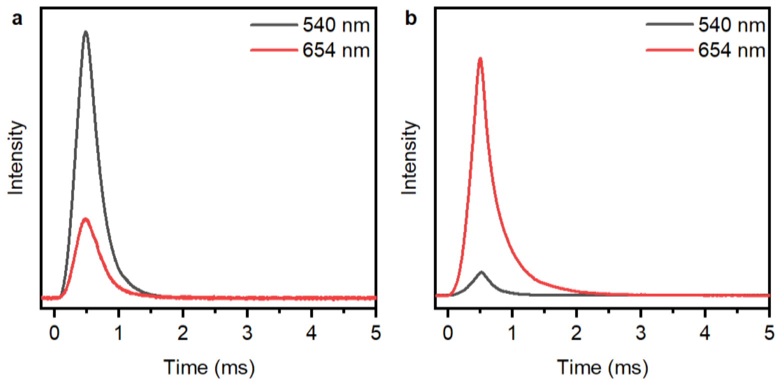

Suppl. Fig. 38 Time-dependent emission intensity of Er^{3+} at 540 and 653 nm from **a** $NaYF_4$:Yb/Er(20/2 mol%)@$NaYF_4$ and **b** $NaErF_4$@$NaYF_4$ nanoparticles. The pulse width under 980 nm excitation is 0.5 ms.

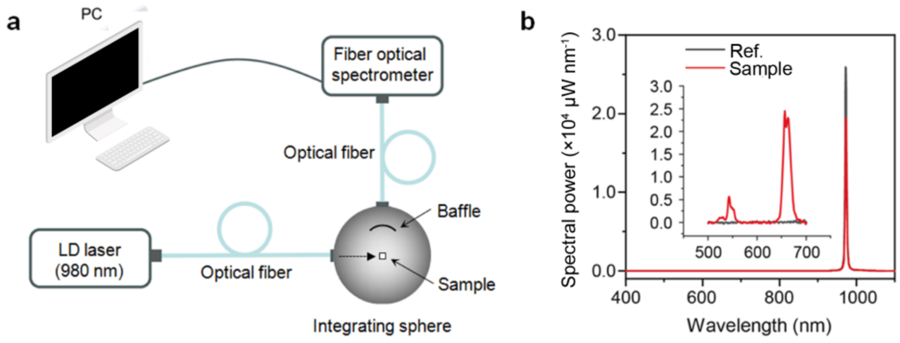

Suppl. Fig. 39 Measurement of upconverison quantum yield. a Schematic of the setup for quantum yield measurement. **b** Measured excitation and emission power spectra from the $NaErF_4$:Ho(0.5 mol%)$NaYbF_4$@$NaYF_4$ core-shell-shell sample and the ref sample under 980 nm excitation (11.6 W cm^{-2}). The reference sample was cyclohexane solution without nanoparticles.

Suppl. Table 1 Lifetime values of Er^{3+} from $NaErF_4$:Ho(0.5 mol%)@$NaYF_4$:Yb(0-100 mol%)@$NaYF_4$ core-shell-shell nanoparticles under pulse 980 nm excitation.

Concentration of Yb^{3+} (mol%)	Lifetime (ms)	
	540 nm	653 nm
0	0.443	0.788
1	0.441	1.032
2	0.596	1.109
5	0.614	1.262
10	0.359	1.112
20	0.323	1.029
60	0.301	0.768
100	0.301	0.704

Suppl. Table 2 Lifetime values of Er^{3+} from $NaErF_4$:Ho(0.5 mol%)@$NaYbF_4$ @$NaYF_4$ core-shell-shell nanoparticles with different $NaYbF_4$ layer thicknesses from 0 to 7.5 nm under pulse 980 nm excitation.

Thickness of $NaYbF_4$ layer (nm)	Lifetime (ms)	
	540 nm	653 nm
0	0.404	0.616
0.4	0.460	0.718
1.0	0.451	0.732
1.8	0.418	0.857
3.1	0.398	0.817
7.5	0.311	0.703

Suppl. Table 3 Lifetime values of Er^{3+} from $NaErF_4$:Ho(0.5 mol%)@$NaYF_4$@ $NaYbF_4$@$NaYF_4$ core-multishell nanoparticles with different $NaYF_4$ interlayer thicknesses from 0 to 8.7 nm under pulse 980 nm excitation.

Thickness of $NaYF_4$ interlayer (nm)	Lifetime (ms)	
	540 nm	653 nm
0	0.292	0.415
0.5	0.306	0.429
1.1	0.387	0.619
2.1	0.464	0.644
4.3	0.427	0.680
8.7	0.335	0.652

Suppl. Table 4 CIE chromaticity coordinates of the visible upconversion emission profiles from the $NaErF_4$:Ho(0.5 mol%)@$NaYF_4$:Yb(20 mol%)@$NaYF_4$ under 980 nm excitation with different power densities.

Pump power density (W cm^{-2})	CIE *x*	CIE *y*
0.82	0.583	0.411
11.60	0.436	0.550
20.83	0.404	0.581
51.62	0.364	0.619
82.41	0.350	0.632
103.93	0.342	0.638

Suppl. Table 5 A summary of the color-tuning properties of the samples used in the preparation of QR pattern.

Samples	Emission colours		
	Long width	Short width	High power
$NaYF_4$:Yb/Er(20/2 mol%)@$NaYF_4$	Green	Green	Green
$NaErF_4$:Ho(0.5 mol%)@$NaYF_4$:Yb(20 mol%)@$NaYF_4$	Red	Green	Green
$NaErF_4$:Ho(0.5 mol%)@$NaYF_4$	Red	Red	Red
$NaYbF_4$:Er(1 mol%)@$NaYF_4$	Green	Green	Red

Suppl. Table 6 A summary of rotation rate, frequency, linear speed and angular speed for the velocity monitoring experiment.

Rotation rate (rev min^{-1})	Rotation frequency (Hz)	Linear speed (km h^{-1})	Angular speed (rad s^{-1})
120	2.0	2.3	4.0π
200	3.3	3.7	6.6π
300	5.0	5.7	10π
400	6.7	7.5	13.4π
600	10.0	11.3	20.0π
800	13.3	15.1	26.6π
1000	16.7	18.8	33.4π
1200	20.0	22.6	40π
